# Resource availability, competitor abundance and specialization affect competition among bumblebees

**DOI:** 10.1093/beheco/araf038

**Published:** 2025-05-03

**Authors:** Zhong-Ming Ye, Yong-Deng He, Wen Huang, Xiao-Fang Jin, Pedro J Bergamo, Chun-Feng Yang

**Affiliations:** State Key Laboratory of Plant Diversity and Specialty Crops, Wuhan Botanical Garden, Chinese Academy of Sciences, Wuhan, 430074, China; State Key Laboratory of Plant Diversity and Specialty Crops, Wuhan Botanical Garden, Chinese Academy of Sciences, Wuhan, 430074, China; University of Chinese Academy of Sciences, Beijing, 101408, China; State Key Laboratory of Plant Diversity and Specialty Crops, Wuhan Botanical Garden, Chinese Academy of Sciences, Wuhan, 430074, China; University of Chinese Academy of Sciences, Beijing, 101408, China; College of Life Sciences, Wuhan University, Wuhan, 430072, China; Universidade Estadual Paulista (Unesp), Instituto de Biociências, câmpus Rio Claro, BR-13506900, Rio Claro, Brazil; State Key Laboratory of Plant Diversity and Specialty Crops, Wuhan Botanical Garden, Chinese Academy of Sciences, Wuhan, 430074, China

**Keywords:** coexistence, interspecific competition, intraspecific competition, specialization

## Abstract

The strength of interspecific and intraspecific competition depends on resource availability, competitor abundance, and specialization. Most studies are conducted with single species pairs, constraining our understanding of what drives competition in multispecies communities. We investigated the effect of floral density, competitor abundance, flowering period, and trait specialization (proboscis length) on the potential for interspecific and intraspecific competition between seven common bumblebee species in the Hengduan Mountains of southwest China. We estimated the potential for interspecific and intraspecific competition using resource partitioning indices in plant-bumblebee networks (Müller’s index) over five consecutive years, quantifying changes in floral densities and bumblebee abundance throughout the season. The potential for interspecific competition increased with bumblebee abundance, particularly when floral density was low later in the season. On the other hand, the potential for intraspecific competition increased with abundance of focal bumblebee species and for specialized long-tongued bumblebees, especially when floral density was low. This suggests that resource scarcity regulates species coexistence by limiting abundant species dominance via interspecific competition and intraspecific competition. Our results indicate the importance of intraspecific competition and specialization in maintaining diversity in multispecies communities.

## Teaser

In bumblebee communities, limited floral resources lead to increased competition. Abundant bumblebee species experience stronger interspecific and intraspecific competition, while specialized long-tongued bumblebees face heightened intraspecific competition. These findings illustrate how resource scarcity and species traits shape bumblebee foraging behavior, fostering coexistence and sustaining community diversity.

## Introduction

Competition is one of the main structuring processes of ecological communities (Vellend 2016). The relative strength of interspecific vs. intraspecific competition determines species coexistence by limiting abundant species dominance and their negative impact on rarer species (HilleRisLambers et al. 2012). Factors such as resource availability, competitor abundance, and specialization regulate the balance of interspecific over intraspecific competition (Koffel et al. 2021). However, most studies have focused on species pairs, constraining our understanding of what drives competition in multispecies communities (Saavedra et al. 2017). Such understanding requires monitoring resource use of multiple species across gradients of resource availability and competitor abundance. In this context, plant-pollinator communities are good models, as it is relatively straightforward to monitor changes in resource availability and competitor abundance throughout the season in these systems (Souza et al. 2018; Sponsler et al. 2023). Moreover, pollinators are regulated by similar foraging constraints as other animal groups (Pyke and Starr 2021), making it possible to investigate common factors determining interspecific vs. intraspecific competition.

Within the same trophic level, species sharing more interaction partners may be more likely to influence each other through indirect effects (Morris et al. 2004). The potential for one species to indirectly influence another species from the same trophic level via shared interaction partners will depend not only on the presence but also on the strength (ie frequency) of each interaction link (Müller et al. 1999; Carvalheiro et al. 2014). For plant–pollinator networks, studies on indirect effects between species of the same trophic level have focused more on the effect among plants sharing pollinators (Carvalheiro et al. 2014; Tur et al. 2016; Bergamo et al. 2021), while relatively fewer studies have focused on indirect effects between pollinator species (Maglianesi et al. 2014; Bendel et al. 2019; Gómez-Martínez et al. 2020). Indirect interactions among pollinators are not restricted to pollinator species pairs and often involve complex competitive hierarchies among multiple species (Fontaine et al. 2008). Quantifying indirect interactions provides a more comprehensive understanding of species coexistence, as it captures the cascading effects that arise from competition for shared resources (Bascompte and Jordano 2007). Direct interactions alone may overlook these broader community-wide dynamics, leading to an incomplete picture of competitive relationships in complex ecological networks (Tylianakis et al. 2010). However, it is not easy to quantify the relative strength of interspecific and intraspecific competition in diverse ecological networks. The Müller’s index was developed to describe indirect interactions by measuring apparent competition between species that share natural enemies (Müller et al. 1999). It is a versatile index applicable to any species group competing for shared resources provided by another species group (eg Jordán et al. 2024) In this study, we applied the Müller’s index to evaluate the strength of potential interspecific and intraspecific competition (overlap and dominance of shared resource use) between bumblebee species sharing floral resources.

As one of the most ubiquitous pollinators, bumblebees are important in both natural and agricultural systems (Hegland and Totland 2008; Garratt et al. 2014). An increasing number of studies have suggested that bumblebee populations are declining (Goulson et al. 2008; Williams et al. 2009a; Roulston and Goodell 2011). A main cause of bumblebee decline is the decrease in floral resources, due to the strict relationship between the availability of floral resources and the diversity and abundance of bumblebees (Roulston and Goodell 2011; Goulson et al. 2015). This is because floral resource availability determines interspecific and intraspecific competition between bumblebees, with consequences for bumblebee community structure (Thomson 2016). Moreover, the competitive ability of bumblebee species should depend on how they monopolize and share floral resources. However, most studies focused on interspecific competition (Morse 1977; Inouye 1978; Walther-Hellwig et al. 2006; Fontaine et al. 2008) while fewer studies focused on intraspecific competition (Heinrich 1979; Gómez-Martínez et al. 2020; Fitzgerald et al. 2022).

In addition to floral resources, bumblebee foraging choices vary with the abundance of competitors (Inouye 1978; Hegland and Totland 2005; Cervantes-Loreto et al. 2021). For instance, removal of one competing bumblebee species rapidly altered the flower resource use of the remaining species (Inouye 1978; Brosi and Briggs 2013). Thus, the foraging patterns of bumblebees are determined by competitive interactions despite potential floral resource preferences. Furthermore, when floral resources are abundant, bumblebees share their preferred floral resources; otherwise, they shift to other available floral resources reducing competition (Pleasants 1981; Fontaine et al. 2008). In general, the most abundant bumblebee species in a community could have a higher potential to influence the foraging patterns of other bumblebees through shared plants, especially if they are generalist species or when they share traits to efficiently exploit the same type of floral resources (Bowers 1985; Goulson and Sparrow 2009). On the other hand, rare species tend to be more specialized and visit fewer plant species that serve as a high-quality food to rear their larvae (Goulson and Darvill 2004), which may increase intraspecific competition. In addition to abundance, traits such as proboscis length also determine bumblebee specialization, in which species with short proboscis visit a wide array of short and open flowers, while species with long proboscis are more restricted to long tubed flowers (Goulson et al. 2005; Inoue and Yokoyama 2006; Bommarco et al. 2012). In this context, competitor abundance and proboscis length may be important factors that regulate interspecific and intraspecific competition between bumblebees.

The coexistence of multiple bumblebee species could be attributed to spatiotemporal heterogeneity in floral resources (Ranta and Vepsäläinen 1981). Therefore, spatial and temporal changes in the abundance and diversity of floral resources and of competitors may influence how bumblebees monopolize and share such resources (Shibata and Kudo 2020). As a consequence, the strength and direction of competitive interactions among bumblebees may fluctuate greatly over the season (Cervantes-Loreto et al. 2021), with potentially stronger competition at the beginning and end of the flowering period when resources are less abundant (Grab et al. 2017; Sponsler et al. 2023). Biodiverse systems present wide spatiotemporal heterogeneity in floral resources, as well as in regulating factors such as abundance and specialization (Bergamo et al. 2020), making them good models to understand interspecific and intraspecific competition between bumblebees.

In this study, we investigated how bumblebees monopolize and share floral resources in a highly biodiverse community. We asked if the strength of potential intraspecific and interspecific competition (measured as overlap weighted by the dominance of shared floral resources) depend on floral density, bumblebee abundance, and bumblebee specialization (proboscis length). We predicted that both potential intraspecific and interspecific competition should increase with the decrease of floral resources. In addition, we expected competition to increase with abundance, as more abundant bumblebee species are more likely to monopolize shared floral resources due to higher foraging pressure and competitive dominance, especially when floral resources are scarcer (Klomp 1964; Koffel et al. 2021). Furthermore, specialized long-tongued bumblebees were expected to exhibit stronger intraspecific competition, in periods of floral resource scarcity.

## Methods

### Study area

We conducted field sampling in an alpine meadow located in Shangri-La County (27°37’40’’ N, 99°47’32’’ E; 3379 m), in the northwestern part of northwest of Yunnan Province, China. The study area was around 12 hectares, a small valley basin surrounded by coniferous forests. The flowering season extends from late June to early September. Approximately 120 entomophilous plant species grow in the meadow, including more than 86 species visited by bumblebees (Ye et al. 2024a).

### Bumblebee survey and specialization

We sampled bumblebees for five consecutive years, from 2018 to 2022. In each survey, we sampled on selected walk transects weekly under warm and dry days (50 m in length and 2 m in width) throughout the flowering season. The transects were placed to include a representative diversity and abundance of floral resources (see details in Appendix S1 in Supporting Information). New transects were added to the field survey to track phenological changes within the meadows. In total, we set up 1420 transects in 38 sampling days across the 5 yr, including 73 transects in 2018 for 5 wk, 140 transects in 2019 for 9 wk, 520 transects in 2020 for 9 wk, 289 transects in 2021 for 8 wk, and 398 transects in 2022 for 7 wk (Table S1). Although sampling spanned June through September, we limited our analysis to July and August, as interaction frequencies were inadequate at the season’s fringes. Additionally, minor adjustments to transect locations were made across years. We recorded bumblebees and the plant species they visited by walking along the transects at constant speed for10 min, from 0930 to 1630 h. Each transect survey was conducted by a two-person team, with one person primarily responsible for capturing bumblebees and the other for recording data. To minimize observer bias, the 5-yr study was led by the two most experienced researchers. During each transect survey, most of the bumblebees were captured and temporarily held in an ice chamber. Identifiable individuals were released immediately after field identification using hand lens (following Williams et al. 2009b; see Ye et al. 2024 for details). Unidentifiable specimens were brought to the lab, where DNA was extracted from a middle leg for COI barcoding (Huang et al. 2015). This approach prevented double-counting individuals within transects. We measured proboscis length (the total length of the prementum and glossa) using a vernier caliper (Cane 1987) as a proxy for trait specialization, based on a total of at least 50 specimens per species across all years. All specimens were deposited in the Wuhan Botanical Garden, Chinese Academy of Sciences.

We constructed daily plant-bumblebee networks, where interaction strength was determined by bumblebee visitation frequency, pooling transect data to ensure adequate network size. We pooled interactions across transects per day to achieve networks of sufficient size. To evaluate the completeness of plant-bumblebee interaction sampling, we pooled all interactions recorded across sampling days within each year to construct annual full networks and then estimated interaction sampling completeness using the Chao 2 estimator, following Chacoff et al. (2012). To determine whether sampling effort (ie the number of transects per year) influenced sampling completeness, we conducted a Spearman correlation analysis between these two variables. The results indicated that our sampling completeness was sufficient and showed no significant correlation with sampling effort (see details in Appendix S2 in Supporting Information).

### Floral resource availability

To quantify the availability of floral resources for bumblebees in each sampling day, we established five 2 × 2 m plots within each walk transect at every 10 meters along the transects. We counted the number of flowers of all plant species within the plots. For highly abundant plant species with multiple flowers per individual, we estimated the average number of flowers per inflorescence or branch from 10 individuals and then used inflorescences or branches as a counting unit (Benadi and Pauw 2018). For less abundant species, we counted single flowers in the whole plant. We estimated floral density as the average number of total open floral units/4 m^2^ per sampling day. Although all flowering plant species in the study area were surveyed, only those visited at least once by a bumblebee were included in plant abundance calculations. Plant species were identified according to Flora of China (Wu et al. 2013). All plant specimens were deposited in the Wuhan Botanical Garden, Chinese Academy of Sciences.

Given the relatively stable flowering season at the study site, typically spanning from June 20 to September 10 each yr, we classified the flowering period into three stages: early (before July 20), middle (July 21–August 20), and late (after August 20). Beyond this general timeline, the classification is further supported by patterns in floral species richness. Analysis of 5 yr of data shows a distinct decline in the number of flowering species around August 20, marking the transition to the late stage. Conversely, floral richness increases around July 20, indicating a shift from the early to middle stage, except in 2021 (Fig. S1). Given the annual variation in the composition of flowering species, we consider this classification the most suitable for this study site.

### Data analysis

We built one plant-bumblebee network per census day per year, totaling 38 quantitative interaction networks. To calculate the potential competition for shared floral resources between bumblebees, we used the Müller’s index (Müller et al. 1999). This is a pairwise quantitative index based on the interaction frequency data that quantifies how much one bumblebee species (“acting species”) affect other (“target species”) by dominating their shared floral resources.

Müller’s index is usually applied in ecological networks to quantify the potential for apparent competition via shared consumers between resource species in antagonistic interaction networks (Müller et al. 1999; Morris et al. 2004), but it is well suited for evaluating the potential for any indirect influence (apparent competition or facilitation, Tack et al. 2011), for example, between plants via shared pollinators (Carvalheiro et al. 2014). In this case, we used the Müller’s index to study potential inter- and intraspecific (with all bumblebee species that co-exist in time and space) competition via the shared plant species on which they feed. Therefore, we transposed the standardized matrices placing the bumblebee species (higher trophic level) in rows and the plant species (lower trophic level) in columns. The index was applied to all pairs of bumblebee species in the plant–bumblebee network:


$$\begin{array}{l} d_{ij}=\underset{k}{\sum }\;\left[\frac{a_{ik}}{\Sigma_{l}a_{il}}\times \frac{a_{jk}}{\Sigma_{m}a_{mk}}\right] \end{array}$$

In this index, $d_{ij}$ is the indirect effect of bumblebee species *j* on bumblebee species *i*; $a_{ik}$ is the number of interactions of the target bumblebee species *i* with all *k* shared plant species with the acting bumblebee *j*. This value is divided by $a_{il}$, being the total number of interactions performed by the target bumblebee *i* with all *l* plants in the network. Correspondingly, $a_{jk}$ represents the number of interactions of the acting bumblebee species *j* with all *k* shared plant species with the target bumblebee *i*. This value is divided by $a_{mk}$ which represents the total number of interactions that *k* shared plants perform with all *m* bumblebees in the network.

The index varies from 0 (if a given bumblebee pair does not share plants) to 1 (maximum effect of the acting bumblebee species on the target bumblebee species by dominating their shared plants). Therefore, a higher value of Müller’s index indicates a greater potential interspecific competition between the acting bumblebee species and the target bumblebee species via shared plants. It produces asymmetric effects among pairs of bumblebee species if each bumblebee species contributes differently to the visits of each plant, as is expected for any species pair in nature. The Müller’s index also quantifies the potential intraspecific competition using the same rationale. In this case, the index quantifies how much each focal bumblebee species contributes to the total visitation of shared plant species. Therefore, bumblebee species that monopolize shared plants are expected to be under stronger intraspecific competition (Müller et al. 1999). The Müller’s index has been used in plant-pollinator networks to quantify plant competition for pollinators (Bergamo et al. 2021), as well as bumblebee competition for floral resources (Gómez-Martínez et al. 2020).

To calculate the Müller’s index, we ran the function PAC of the bipartite R-package for each of the 38 plant-bumblebee networks separately (Dormann et al. 2008). The outcome of the PAC function for each network was a k × k matrix with the same bumblebee species in rows and columns. Values for each cell represent the potential influence of the species in the column on the species in the row through shared feeding plants. For each plant-bumblebee network per census day, we averaged the values by column (not counting the ones in the diagonal), obtaining the mean effect that the focal bumblebee species (acting species) had on the rest of the species in the network, which represent the potential interspecific competition (Carvalheiro et al. 2014; Gómez-Martínez et al. 2020). Diagonal values estimate the extent of potential intraspecific competition via shared plants (Dormann et al. 2008).

Since bumblebee abundance was recorded as the number of individuals caught interacting with flowers, there is an inherent circularity between bumblebee abundance and the interaction frequencies used to construct the daily plant-bumblebee networks. To circumvent this, we estimated bumblebee abundance from quantitative interaction data using the mass action principle (Staniczenko et al. 2013). The mass action principle originates from chemistry, where it states that the rate of a chemical reaction is proportional to the product of the concentrations or masses of the reactants. In an ecological context, it assumes that the interaction frequency ($F_{ij}$) between species is proportional to the product of their relative abundances ($x_{i}$ = abundance of pollinator species i; $x_{j}$ = abundance of plant species j) and their interaction preference ($c_{ij}$), such that $F_{ij}$ = $x_{i}\;\;\;x_{j}\;\;\;c_{ij}$. Therefore, when sufficient interaction frequency data ($F_{ij}$) is available, it is possible to infer the interaction preference ($c_{ij}$) and then the effective relative abundances of the interaction partners ($x_{i}$ and $x_{j}$) (Staniczenko et al. 2013). We then used this estimated abundance in all statistical models to diminish a potential circularity between abundance and Müller’s index.

To test the effects of floral density, bumblebee abundance, and specialization (proboscis length) on potential interspecific competition, we used the average interspecific Müller’s index values of each of the 38 interaction networks, which were sampled throughout the 5 yr as the response variable, in a Generalized Linear Mixed Model (GLMM) using a Gaussian distribution to meet model assumptions. Floral density on the census date, estimated abundance of acting bumblebee species on the census date, proboscis length, flowering period, and year were used as fixed effects. We also included the interaction between floral density and abundance of the acting species to test the prediction that abundant bumblebees compete more for resources at lower floral densities. We did not include the interaction between proboscis length and floral density because we used a single trait value per bumblebee species and thus, proboscis length is fixed across the census. The identity of the acting bumblebee species was included as random effects.

To test the effects of floral density, bumblebee abundance, and specialization (proboscis length) on potential intraspecific competition, we used the intraspecific Müller’s index as the response variable in a Generalized Linear Mixed Model (GLMM) using Gaussian distribution to meet model assumptions. Floral density, abundance of the focal bumblebee species, proboscis length of the focal bumblebee species, year, flowering period (early, middle or late), and the interaction between floral density and bumblebee abundance were used as fixed effects. The identification of the focal bumblebee species was included as random effects.

The values of floral density and bumblebee abundance were log- and z-transformed (scaled to a mean of 0 and standard deviation of 1) to improve model convergence. The proboscis length was only z-transformed. All factors had Variation Inflation Factor (VIF)  < 3 in the models, and thus we assumed robustness to multicollinearity (Zuur et al. 2010). Model diagnostics were conducted using the simulateResiduals function from the DHARMa package to assess compliance with distributional assumptions. Residual uniformity was formally tested via a Kolmogorov-Smirnov test (interspecific model: D = 0.08, p = 0.195; intraspecific model: D = 0.05, p = 0.647), while dispersion patterns were evaluated through simulated versus observed residual variance (interspecific model: dispersion ratio = 1.19, p = 0.36; intraspecific model: dispersion ratio = 1.08, p = 0.536). Both tests confirmed the adequacy of the models’ assumptions. We plotted partial residuals of each variable included in the best model using the visreg R-package (Breheny and Burchett 2017). Generalized Linear Mixed Models (GLMMs) were performed using the glmmTMB package (Brooks et al. 2017). Wald’s chi-square tests for each GLMM were performed using “Anova” in car package (Fox and Weisberg 2019).

Because the sampling effort (number of walked transects per census day) of 2018 and 2019 were lower than the other 3 yr (2020–2022), we redid both GLMMs for potential for interspecific and intraspecific competition only using the data from 2020 to 2022 to detect whether the sampling effort affected the results.

All data analysis were performed in R 4.2.2 (R Core Team 2022).

## Results

In total, 10598 bumblebees belonging to 14 species were recorded foraging flowers during 5 yr on 38 census days and 1470 walking transects (Table S1). These 14 species account for 70% of the 20 bumblebee species previously documented in the Shangri-La region, the alpine meadow area in northwestern Yunnan (Liang et al. 2018). Among the 14 recorded *Bombus* species, *B. friseanus* (5048, 47.63%), *B. lepidus* (3172, 29.93%), and *B. festivus* (1607, 15.16%) were the most dominant ones; followed by *B. impetuosus* (477, 4.50%), *B. securus* (131, 1.24%), *B. minshanicola* (78, 0.74%), *B. nobilis* (48, 0.45%). Furthermore, *B. prshewalskyi* (13, 0.12%), *B. graham* (7, 0.07%), *B. avanus* (6, 0.06%), *B. hengduanensis* (6, 0.06%), *B. remotus* (3, 0.03%), *B. infrequens* (1, 0.01%) and *B. turneri* (1, 0.01%) were rarely recorded, and thus we excluded them from the analyses of potential for interspecific and intraspecific competition. The average proboscis length per species ranged from 6.60 mm (*B. lepidus*) to 14.24 mm (*B. securus*) (Table S2). There were only two species with proboscis lengths greater than 10 mm, *B. securus* (14.24 mm) and *B. nobilis* (12.75 mm).

Throughout the 5-yr survey, we recorded 89 plant species pollinated by bumblebees belonging to 22 families. The plant species visited by the highest number of bumblebee individuals were *Phlomoides atropurpurea* (36.45%), *Bistorta macrophylla* (20.62%), *Pedicularis siphonantha* (6.61%), and *Ligularia pleurocaulis* (5.89%). The most species-rich plant families were Asteraceae (18), Orobanchaceae (8), Rosaceae (8), Lamiaceae (7), Ranunculaceae (7), and Fabaceae (6).

**Fig. 1.**
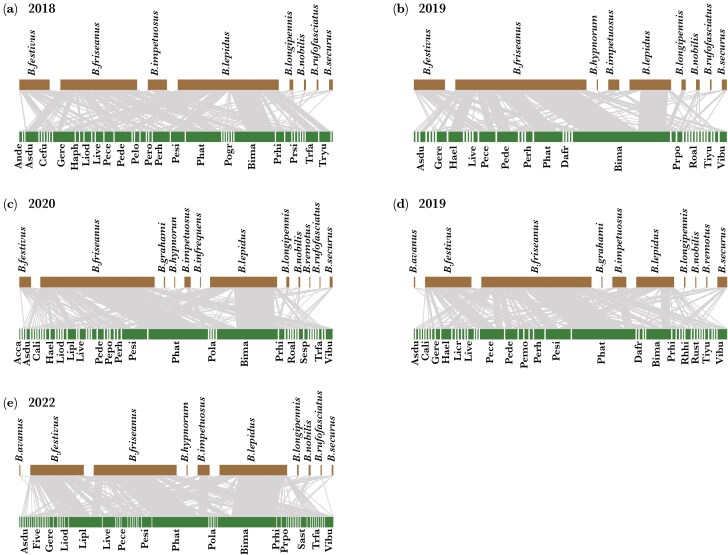
Plant-bumblebee networks from 2018 (a) to 2022 (e). Above (brown): bumblebee species and below (green): plant species. Grey links indicated the frequency of interactions. Each block represents a species, its size is proportional to the total number of interactions, line width shows the abundance of interactions between the two linked partners. The plant species information represented by the abbreviation is as follows: *Aconitum carmichaelii* (Acca), *Allium prattii* (Alpr), *Anemone demissa* (Ande), *Astragalus dumetorum* (Asdu), *Bistorta macrophylla* (Bima), *Carpesium lipskyi* (Cali), *Cerastium furcatum* (Cefu), *Dasiphora fruticosa* (Dafr), *Euphrasia regelii* (Eure), *Filipendula vestita* (Five), *Gentiana haynaldii* (Geha), *Geranium refractum* (Gere), *Halenia elliptica* (Hael), *Hansenia phaea* (Haph), *Iris bulleyana* (Irbu), *Ligularia cremanthodioides* (Licr), *Ligularia odontomanes* (Liod), *Ligularia pleurocaulis* (Lipl), *Ligularia vellerea* (Live), *Parasenecio forrestii* (Pafo), *Pedicularis cephalantha* (Pece), *Pedicularis densispica* (Pede), *Pedicularis longiflora* (Pelo), *Pedicularis monbeigiana* (Pemo), *Pedicularis polyodonta* (Pepo), *Pedicularis rex* (Pere), *Pedicularis rhinanthoides* (Perh), *Pedicularis siphonantha* (Pesi), *Phlomoides atropurpurea* (Phat), *Potentilla griffithii* (Pogr), *Potentilla lancinate* (Pola), *Prunella hispida* (Prhi), *Primula poissonii* (Prpo), *Primula sikkimensis* (Prsi), *Rhododendron hippophaeoides* (Rhhi), *Roscoea alpina* (Roal), *Rubus stans* (Rust), *Saussurea dolichopoda* (Sado), *Salvia flava* (Safl), *Saussurea stella* (Sast), *Senecio spathiphyllus* (Sesp), *Taraxacum mongolicum* (Tamo), *Tibetia yunnanensis* (Tiyu), *Trollius farreri* (Trfa), *Trollius yunnanensis* (Tryu) and *Vicia bungei* (Vibu).

There were 245 links (pairs of interacting species) across the plant-bumblebee interaction networks over the 5 yr. Of the 245 links, 120 (48.98%) were observed in only 1 yr, 40 (16.33%) in 2 yr, 29 (11.84%) in 3 yr, 26 (10.61%) in 4 yr, and 30 (12.24%) in all 5 yr. The interaction frequencies were also highly uneven for each year: few interactions were highly frequent, and most were infrequent (Fig. 1a-e). The number of flowering plant species ranged from 36 to 64 species throughout the 5 yr, with a coefficient of variation of 23.08%; the number of bumblebee species ranged from 8 to 12, with a coefficient of variation of 15.14%; and the number of links ranged from 72 to 145, with a coefficient of variation of 25.67%.

**Fig. 2.**
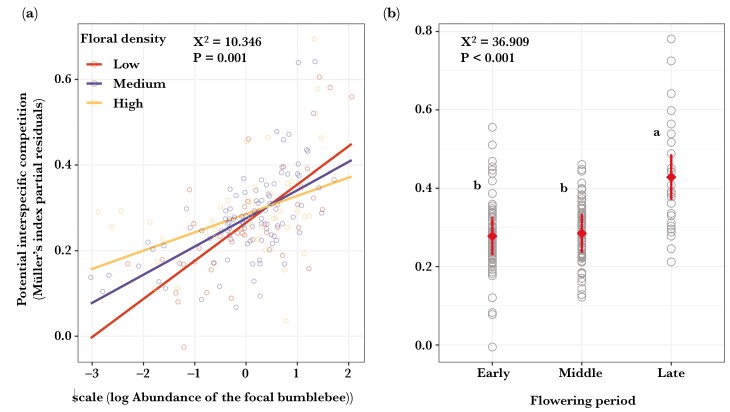
Partial residuals plots showing the predicted values of **(a)** the interactive effect between abundance of the focal species with floral density, for low- (10th quantile; red dots and lines), medium- (50th quantile; blue dots and lines) and high (90th quantile; yellow dots and lines) floral density, and **(b)** the effect of flowering period (early, middle and late) on the potential interspecific competition. The average Müller’s index of acting bumblebee species for interspecific competition was used as the response variable. Floral density (n floral units/4 m^2^) and abundance of the acting bumblebee (n individuals) were log(x)- and z-transformed. For details on model estimates and significance, see Table 1.

The potential for interspecific competition was influenced by abundance of the acting bumblebee species and its interaction effects with floral density (Table 1). The strength of the potential for interspecific competition increased with the abundance of the acting species, particularly when floral density was low (Table 1; Fig. 2a). The potential for interspecific competition was not related to proboscis length and year (Table 1). The potential for interspecific competition was more intense during the late flowering period than during the early and middle periods (Table 1; Fig. 2b).

**Table 1.** Results of the model using the data from 2018 to 2022 showing the relationship between floral density, abundance of the acting bumblebee, proboscis length and potential interspecific competition (average interspecific Müller’s index of acting bumblebee species) as the response variable. The acting bumblebee species identity was included as random effects. Floral density and abundance of the acting bumblebee were log(x)- and z-transformed. The proboscis length was only z-transformed. Bold values indicate significant effects at P < 0.05.

Random effect	Variance	Std.Dev.		
Bumblebee species identity	0.005	0.073		
Fixed effect	Estimate	Std.Error	Z value	P value
(Intercept)	0.085	0.035	2.397	**0.017**
Abundance of the acting bumblebee	0.068	0.009	7.188	**<0.001**
Floral density	0.009	0.009	0.976	0.329
Proboscis length	−0.043	0.026	−1.628	0.104
Year (2019)	0.042	0.027	1.575	0.115
Year (2020)	0.024	0.026	0.895	0.371
Year (2021)	0.012	0.026	0.472	0.637
Year (2022)	0.031	0.026	1.222	0.222
Flowering period (Middle)	0.007	0.016	0.476	0.634
Flowering period (Late)	0.151	0.026	5.851	**<0.001**
Abundance of the acting bumblebee * Floral density	−0.020	0.006	−3.217	**0.001**

**Fig. 3.**
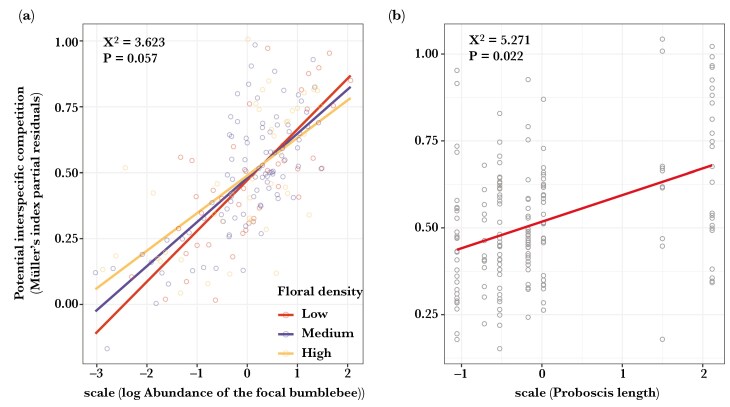
Partial residuals plots showing the predicted values of (a) the interactive effect of abundance of the focal species and floral density, for low- (10th quantile; red dots and lines), medium- (50th quantile; blue dots and lines) and high (90th quantile; yellow dots and lines) floral density, and (b) proboscis length on the potential intraspecific competition. The Müller’s index for intraspecific competition was used as the response variable. Floral density (n floral units/4 m^2^) and abundance of the focal bumblebee (n individuals) were log(x)- and z-transformed. The proboscis length (in mm) was only z-transformed. For details on model estimates and significance, see Table 2.

The potential for intraspecific competition was affected by the abundance of the bumblebee species, with a marginally significant interaction effect with floral density (Table 2). The potential for intraspecific competition increased with the abundance of the focal bumblebee (Table 2; Fig. 3a), especially when floral density was low (Table 2; Fig. 3b). Furthermore, the potential for intraspecific competition increased with the proboscis length of the focal bumblebee (Table 2; Fig. 3c). The flowering period had no significant effect on the potential for intraspecific competition (Table 2).

**Table 2.** Results of the model using the data from 2018 to 2022 showing the relationship between the floral density, abundance of the focal bumblebee, proboscis length and potential intraspecific competition with the Müller’s index values (diagonal values of the PAC matrix) as the response variable. Focal bumblebee species identity was included as random effects. Floral density and abundance of the focal bumblebee were log(x)- and z-transformed. The proboscis length was only z-transformed. Bold values indicate significant effects at P < 0.05 or marginal significance.

Random effect	Variance	Std.Dev.		
Bumblebee species identity	0.008	0.088		
Fixed effect	Estimate	Std.Error	Z value	P value
(Intercept)	0.477	0.052	9.246	**<0.001**
Abundance of the focal bumblebee	0.170	0.017	9.991	**<0.001**
Floral density	0.008	0.016	0.468	0.640
Proboscis length	0.077	0.033	2.296	**0.022**
Year (2019)	0.034	0.049	0.704	0.482
Year (2020)	−0.068	0.048	−1.415	0.157
Year (2021)	−0.002	0.047	−0.042	0.966
Year (2022)	−0.037	0.047	−0.800	0.424
Flowering period (Middle)	0.004	0.028	0.126	0.900
Flowering period (Late)	0.008	0.047	0.177	0.859
Abundance of the focal bumblebee * Floral density	−0.022	0.011	−1.903	**0.057**

The results of GLMMs for the potential for interspecific and intraspecific competition only using data from 2020 to 2022 (the years more intensely sampled) were also qualitatively similar to those using the complete data set (2018–2022) (Tables S3, S4; Figs S2, S3). This indicates that the lower sampling effort (number of walked transects per census day) of 2018 and 2019 did not qualitatively affect the results.

## Discussion

Our study revealed that the potential for interspecific competition increased with bumblebee abundance depending on resource availability, particularly when floral density was low. The potential for intraspecific competition also increased with the abundance of focal bumblebee species, especially when floral density was low. Moreover, intraspecific competition was stronger within specialized long-tongued bumblebee species. Overall, interspecific competition and intraspecific competition depended on bumblebee abundance; such relationship was regulated by floral resource levels in our study system.

The importance of competition in shaping bumblebee communities has long been emphasized (Heinrich 1976; Obeso 1992). Periods of high floral availability increase the possibilities for pollinators to focus on different floral resources and avoid competition (Westphal et al. 2006; Lopes et al. 2022). The potential for interspecific competition increased with the abundance of the acting bumblebee species (ie the ones that most dominated shared floral resources, Gómez-Martínez et al. 2020). The competitive effect of abundant species was stronger when floral resources were scarce and thus, when there were reduced opportunities to switch to distinct floral resources. In addition, interspecific competition was more intense during the late flowering period compared to the early and middle periods, likely due to the decline in floral resource availability at the end of the flowering season (Grab et al. 2017; Sponsler et al. 2023). This suggests that seasonal changes in floral density further regulate interspecific competition, with bumblebees facing increased competition as floral resources decrease. Multiple bumblebee species can co-exist if some species can flexibly change their floral resources in response to the abundance of competitors (Shibata and Kudo 2020). On the other hand, the strength of interspecific competition was not related to proboscis length in this study. Resource partitioning mediated by proboscis length has long been thought to be an important process in allowing bumblebee species coexistence (Heinrich 1976; Inouye 1978; Pyke 1982; Harder 1985; Johnson 1986; Graham and Jones 1996). This lack of relationship may be due to interspecific overlap in proboscis length caused by a wide intraspecific variation in this trait (Table S2). Most of the bumblebee species exhibited shorter proboscis (6.60 to 9.19 mm) and were generalists, visiting several plant species, indicating that interspecific competition among such generalist bumblebee species was not evident in our study community. Goulson et al. (2005) classified bumblebee species into three categories according to their proboscis length: short (shorter than 8 mm), medium (between 8 and 9 mm), and long (longer than 9 mm). Other studies have also failed to find a relationship between variation in proboscis length and bumblebee co-cooccurrence (Ranta 1982; Ranta and Tiainen 1982; Williams 1989). Overall, our results showed that resource availability and competitor abundance are more important than trait specialization in mediating interspecific competition in this system.

Previous studies in bumblebee communities focused on interspecific resource competition while neglecting intraspecific competition (but see Gómez-Martínez et al. 2020). Our results showed that the intensity of intraspecific competition increased with the abundance of the focal bumblebee species, and tended to be stronger when floral resources were scarce. This pattern is in line with theoretical expectations of stronger intraspecific competition when more individuals of the same species simultaneously demand use of a limited resource, and competition increases with the limitation of such resources (Klomp 1964; Koffel et al. 2021). Thus, intraspecific competition may play a key role in reducing the dominance of abundant bumblebees, allowing the persistence of rarer species in critical periods of resource scarcity (Benadi 2015). Moreover, intraspecific competition can lead to the exclusion of subordinate colonies and selection of the fittest ones. Since reproductive success in bumblebees is closely linked to resource availability, competition for floral resources can directly impact colony growth and reproduction (Pelletier and McNeil 2003). Thus, it may impact ecological and evolutionary processes, contributing to trait selection (Bolnick et al. 2011). In addition, intraspecific competition was stronger among bumblebees with long proboscis. This is because bumblebee species with long proboscis usually are more specialized in their use of floral resources, concentrating visits on long tubed flowers (Ranta and Lundberg 1980; Inoue and Yokoyama 2006). On the other hand, short proboscis generalists have more foraging options, thus their individuals can specialize in distinct floral resources and suffer less intraspecific competition (Tur et al. 2015; Liang et al. 2021). In our survey, we recorded only 14 long-tubed plant species (corolla tube or spur length > 10 mm), but several short-tubed (0 to 10 mm) and open flowers (41 and 32 species, respectively), potentially intensifying resource competition among long-proboscis bumblebees. Unlike interspecific competition, however, intraspecific competition was not influenced by flowering period, suggesting that the factors driving intraspecific competition remain consistent throughout the flowering season. Thus, intraspecific competition may respond more to fine-scale floral resource dynamics than to flowering phenology changes at the community-level. In sum, we found that intraspecific competition played a potential role in promoting bumblebee coexistence by potentially regulating abundant species.

Interspecific and intraspecific competition act together to shape ecological communities. In a highly biodiverse bumblebee community, we extensively monitored changes in resource availability, competitor abundance, and a gradient of specialization (proboscis length) to understand the drivers of potential competition. Our results indicated that resource scarcity may regulate bumblebee coexistence by limiting abundant species dominance through interspecific and intraspecific competition. Abundant and short proboscis generalists likely replaced each other in dominating shared floral resources throughout the season, resulting in no relationship between proboscis length and potential interspecific competition. On the other hand, long proboscis specialists were consistently under stronger potential intraspecific competition, illustrating the link between trait specialization and competition. Our results highlight the importance of intraspecific competition and how resource availability and specialization regulate its role in maintaining diversity in multispecies communities.

## Supplementary Material

araf038_suppl_Supplementary_Materials

## Data Availability

Analyses reported in this article can be reproduced using the data provided by Ye et al. (2024b, 2025).
