# Supplementary material for: Resource availability, competitor abundance and specialization affect competition among bumblebees
Source: Behav Ecol. 2025 May 3;36(4):araf038. doi: 10.1093/beheco/araf038 (PMC12130794; doi:10.1093/beheco/araf038)
Supplement: araf038_suppl_Supplementary_Materials [file araf038_suppl_supplementary_materials.pdf]

Supporting Information

Title: Resource availability, competitor abundance and specialization affect competition among bumblebees

Appendix S1: The details of sampling methods

Appendix S2: Interaction sampling completeness

Table S1: The number of transects and individuals for each bumblebee species for each census day for each year.

Table S2: The descriptive statistics of the proboscis length of the seven most common species.

Table S3: Results of the Gaussian GLMM for potential interspecific competition using only data from 2020 to 2022.

Table S4: Results of the Gaussian GLMM for potential intraspecific competition only using the data from 2020 to 2022.

Fig. S1: Plant richness (the number of flowering species) recorded on each census day from 2018 to 2022.

Fig. S2: Partial residuals plots showing the predicted values on the strength of potential interspecific competition by only using the data from 2020 to 2022.

Fig. S3: Partial residuals plots showing the predicted values on the strength of potential intraspecific competition by only using the data from 2020 to 2022.

Appendix S1. The details of sampling methods

The study area was around 12 hectares, a small valley basin surrounded by mountains (Fig. A1 A). In this meadow, there were three types of habitat with different dominant species. Type 1 was the forest edge (Fig. A1 B) with the transects indicated by green lines in Fig. A1 A. Type 2 was the wet meadow (Fig. A1 C) and the transects indicated by purple lines. Type 3 was the dry meadow (Fig. A1 D) and the transects indicated by red lines. The area of Type 1, 2 and 3 was 3,3.5 and 5.5 hectares, respectively. The Type 1 habitat was usually located along the forest, the Type 2 habitat was usually located on the high land with dry soil, while the Type 2 habitat was usually located on the low land or the ditch with wet soil. The dominant plants in the three regions were different, but the distribution of plants within each habitat was relatively uniform. In forest edge habitat (Type 1), the dominant plant species were *Trollius yunnanensis*, *Primula sikkimensis*, *Pedicularis monbeigiana*, *Primula secundiflora*, *Anemone rivularis*, *Astragalus dumetorum* and *Ligusticopsis brachyloba*. In wet meadow habitat (Type 2), the dominant plant species were *Phlomoides atropurpurea*, *Primula poissonii*, *Pedicularis cephalantha*, *Ranunculus* L., *Trollius farreri*, *Anemone rupestris*, *Pedicularis rhinanthoides, Halenia elliptica,* and *Pedicularis densispica*. In dry meadow habitat (Type 3), the dominant plant species were *Bistorta macrophylla*, *Potentilla lancinata*, *Vicia bungei*, *Pedicularis siphonantha*, *Ligularia pleurocaulis*, *Ligularia vellerea*, *Pedicularis polyodonta*, and *Senecio spathiphyllus*. Some plant species that existed occurred in two or three areas; after all, there was no absolute isolation boundary between the habitats.

To ensure representative sampling, we employed a random transect placement approach within each habitat type. We then adjusted the number of transects for each habitat to match its respective area. For instance, we assigned 8, 11, and 16 transects

to each habitat type in 2021, as shown in Fig. A1 A. The ratio of the number of transects between the habitats was not much different from the proportion of their area. This method allowed us to cover the diverse range of flowering plants in the community in a comprehensive way, while avoiding localized sampling bias. In 2018 and 2019, at the beginning of the flowering season, when plant richness and abundance were relatively low, we used a smaller number of transects. As the season progressed and the floral diversity increased, we added new transects to encompass emerging plants. In 2020-2022, we increased the number of transects to enhance the intensity of sampling (see Table S1). For these final years of sampling, we had gained insight into the timing and occurrence of various plants throughout the meadow. Consequently, we were able to establish all transects at the beginning of the season, resulting in minimal variation in transect numbers between survey days. Each transect was assigned a unique ID and remained consistent throughout the entire season within the respective year. Although many transects were placed in similar locations over the five-year study period, we did not maintain the same IDs across years, as our study design did not aim to emphasize year-to-year replication at the transect level.

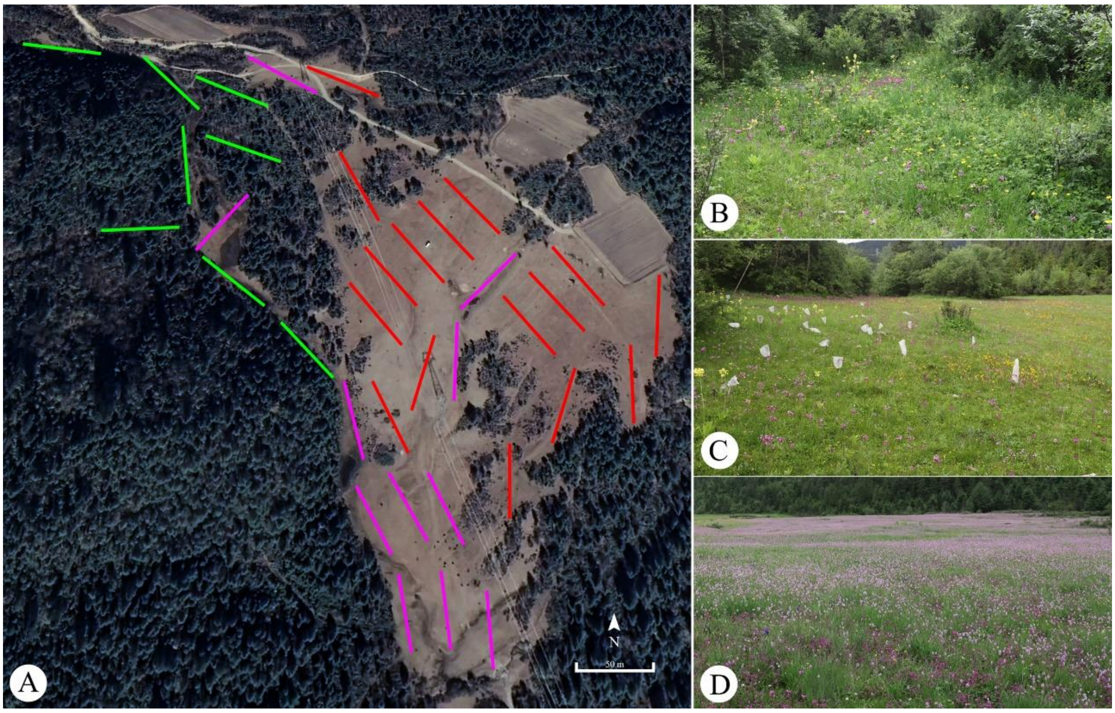

Fig. A1. The distribution of the sampling transects and the appearance of the habitat of three types. The distribution of sampling transects in 2021 (A), which belong to Type 1, 2 and 3 were labelled with green, purple, and red lines, respectively; the habitat appearance of forest edge (B; Type 1); the habitat appearance of wet meadow (C; Type 2); the habitat appearance of dry meadow (D; Type 3).

Appendix S2. Interaction sampling completeness

We pooled all interactions sampled across sampling days per year to obtain a plant-bumblebee visitation network per year. We estimated the sampling completeness of plant-bumblebee interactions in the whole network per year following Chacoff et al. (2012), as:

$$\%S = \frac{S_O}{S_E}$$

in which S_O is the observed richness of interactions observed in each visitation network, and S_E is the asymptotic interaction richness estimated with Chao 2. To determine whether there was any influence of sampling effort (number of transects per year) on interaction sampling completeness per year, we used a Spearman correlation to test their relationship. We estimated Chao 2 using the R package iNEXT (Hsieh et al. 2016).

The sampling completeness of plant-bumblebee interaction showed that 74.60% of the interactions were sampled in 2018, 34.50% in 2019, 79.24% in 2020, 63.24% in 2021, and 65.88% in 2022 (Table A1). Overall, our sampling completeness was quite high, except for 2019, which was relatively low. We inferred that the relatively lower sampling completeness in 2019 (34.50%) may have resulted from a combination of factors, particularly lower rainfall that year, which led to reduced floral availability and subsequently lower bumblebee abundance. In contrast, although the number of transects in 2018 was even lower than in 2019, the higher bumblebee abundance in 2018 contributed to a relatively high sampling completeness that year. Although the sampling effort differed between years, interaction sampling completeness was not correlated with the sampling effort (Spearman correlation: $r = 0.40$, $n = 5$, $P = 0.52$).

Table A1. The sampling completeness of each year. S_O is the observed richness of interactions observed in each visitation network and S_E is the asymptotic interaction richness estimated with Chao 2.

Year	S_O	S_E	Sampling completeness
2018	94	125.994	74.60%
2019	72	208.686	34.50%
2020	123	155.229	79.24%
2021	107	169.182	63.24%
2022	145	220.091	65.88%

References

Chacoff NP, Vázquez DP, Lomáscolo SB, Stevani EL, Dorado J, Padrón B. (2012). Evaluating sampling completeness in a desert plant–pollinator network. J. Anim. Ecol. 81: 190-200. https://doi.org/10.1111/j.1365-2656.2011.01883.x

Hsieh TC, Ma KH, Chao A. 2016. iNEXT: an R package for rarefaction and extrapolation of species diversity (Hill numbers). Methods Ecol. Evol. 7: 1451–1456. https://doi.org/10.1111/2041-210X.12613

Table S1. The number of transects and individuals of each bumblebee species for each census day for each year. The asterisks mean the transects were observed twice on this census day. The species information represented by the abbreviation (in the brackets) is as follows: *B. friseanus* (Fri), *B. lepidus* (Lep), *B. festivus* (Fes), *B. impetuosus* (Imp), *B. secures* (Sec), *B. minshanicola* (Min), *B. nobilis* (Nob), *B. prshewalskyi* (Prs), *B. graham* (Gra), *B. avanus* (Ava), *B. hengduanensis* (Hen), *B. remotus* (Rem), *B. infrequens* (Inf) and *B. turneri* (Tur).

Year	Date	Transects	Total	Fri	Lep	Fes	Imp	Sec	Min	Nob	Prs	Gra	Ava	Hen	Rem	Inf	Tur
2018	20180702	10	56	9	41	3	2	0	0	1	0	0	0	0	0	0	0
	20180708	13*	205	61	107	21	7	3	4	1	1	0	0	0	0	0	0
	20180714	15	133	42	56	9	14	7	2	2	1	0	0	0	0	0	0
	20180721	21	240	86	84	45	21	0	3	0	1	0	0	0	0	0	0
	20180728	14	113	52	24	19	17	0	1	0	0	0	0	0	0	0	0
2019	20190704	12	46	7	30	1	5	1	1	0	0	0	0	1	0	0	0
	20190710	14*	174	88	43	26	6	5	3	3	0	0	0	0	0	0	0
	20190716	16	80	50	15	9	2	1	0	2	1	0	0	0	0	0	0
	20190722	18	98	56	15	18	5	0	3	1	0	0	0	0	0	0	0
	20190729	19	68	40	13	6	3	2	3	0	1	0	0	0	0	0	0

	20190811	20	41	26	1	12	0	0	0	1	0	0	0	0	0	0	1
	20190817	18	27	18	0	9	0	0	0	0	0	0	0	0	0	0	0
	20190823	14*	35	30	0	2	3	0	0	0	0	0	0	0	0	0	0
	20190906	9*	44	38	0	3	2	1	0	0	0	0	0	0	0	0	0
2020	20200623	51	15	2	13	0	0	0	0	0	0	0	0	0	0	0	0
	20200704	59	293	71	213	4	3	2	0	0	0	0	0	0	0	0	0
	20200710	60	629	227	369	20	3	7	2	1	0	0	0	0	0	0	0
	20200718	61	1042	354	586	54	14	11	20	2	0	0	0	1	0	0	0
	20200728	59	962	587	191	106	34	10	20	7	2	4	0	0	1	0	0
	20200811	60	770	680	4	36	37	6	0	7	0	0	0	0	0	0	0
	20200821	58	280	257	3	5	11	2	0	0	0	1	0	0	0	1	0
	20200828	57	134	119	0	1	10	2	1	0	0	1	0	0	0	0	0
	20200907	55	74	67	0	0	5	2	0	0	0	0	0	0	0	0	0
2021	20210704	35	18	3	9	1	0	4	0	0	0	0	1	0	0	0	0
	20210711	35	84	27	41	4	2	10	0	0	0	0	0	0	0	0	0
	20210717	35	206	66	84	35	5	14	1	0	0	0	0	0	1	0	0

	20210724	35	156	60	52	34	5	5	0	0	0	0	0	0	0	0	0
	20210730	35	182	81	22	63	10	5	0	0	0	0	0	0	1	0	0
	20210806	38	182	86	17	43	21	12	1	1	0	1	0	0	0	0	0
	20210814	38	345	224	4	85	28	2	0	0	0	0	2	0	0	0	0
	20210830	38	168	130	1	24	10	3	0	0	0	0	0	0	0	0	0
2022	20220703	56	482	99	354	17	2	3	4	1	2	0	0	0	0	0	0
	20220713	57	573	72	386	98	5	0	2	7	2	0	0	1	0	0	0
	20220722	57	796	179	336	241	25	3	4	6	1	0	0	1	0	0	0
	20220802	57	859	500	47	236	65	3	2	3	0	0	1	2	0	0	0
	20220811	57	691	395	11	214	66	1	1	2	1	0	0	0	0	0	0
	20220819	57	197	80	0	93	23	0	0	0	0	0	1	0	0	0	0
	20220828	57	100	79	0	10	6	4	0	0	0	0	1	0	0	0	0
Total	38	1420	10598	5048	3172	1607	477	131	78	48	13	7	6	6	3	1	1

Table S2. The descriptive statistics of the proboscis length of the most seven common species.

Species	N	Mean	Std. Dev.	Min.	Max.
B. lepidus	1576	6.60	0.67	4.55	9.8
B. friseanus	1937	7.88	0.84	5.1	11.87
B. impetuosus	303	8.73	0.86	6.75	12.09
B. festivus	1136	9.19	0.80	6.27	13.7
B. minshanicola	70	7.42	0.87	5.11	9.39
B. nobilis	59	12.75	1.54	8.96	15.97
B. secures	123	14.24	2.75	9.15	20.58

Table S3. Results of the model by only using the data from 2020 to 2022 showing the relationship between floral density, abundance of the acting bumblebee, proboscis length and potential interspecific competition (average interspecific Müller's index of acting bumblebee species) as the response variable. The acting bumblebee species identity was included as random effects. Floral density and abundance of the acting bumblebee were log(x)- and z-transformed. The proboscis length was only z-transformed. Bold values indicate significant effects at $P < 0.05$.

Random effect	Variance	Std.Dev.		
Bumblebee species identity	0.004	0.059		
**Fixed effect**	**Estimate**	**Std.Error**	**Z value**	**P value**
(Intercept)	0.105	0.029	3.674	**<0.001**
Abundance of the acting bumblebee	0.085	0.012	6.857	**<0.001**
Floral density	0.007	0.012	0.551	0.582
Proboscis length	-0.042	0.024	-1.738	0.082
Year (2021)	-0.009	0.022	-0.425	0.671
Year (2022)	0.006	0.022	0.256	0.798
Flowering period (Middle)	0.008	0.020	0.407	0.684
Flowering period (Late)	0.141	0.035	3.972	**<0.001**
Abundance of the acting bumblebee * Floral density	-0.032	0.008	-3.885	**<0.001**

Table S4. Results of the model by only using the data from 2020 to 2022 showing the relationship between the floral density, abundance of the focal bumblebee, proboscis length and potential interspecific competition with the Müller's index values (diagonal values of the PAC matrix) as the response variable. Focal bumblebee species identity was included as random effects. Floral density and abundance of the focal bumblebee were log(x)- and z-transformed. The proboscis length was only z-transformed. Bold values indicate significant effects at $P < 0.05$.

Random effect	Variance	Std.Dev.		
Bumblebee species identity	0.010	0.099		
Fixed effect	Estimate	Std.Error	Z value	P value
(Intercept)	0.425	0.049	8.682	**<0.001**
Abundance of the focal bumblebee	0.193	0.022	8.846	**<0.001**
Floral density	0.009	0.022	0.415	0.678
Proboscis length	0.106	0.041	2.615	**0.009**
Year (2021)	0.084	0.039	2.135	**0.033**
Year (2022)	0.038	0.040	0.959	0.337
Flowering period (Late)	-0.038	0.035	-1.076	0.282
Flowering period (Middle)	0.014	0.063	0.227	0.821
Abundance of the focal bumblebee * Floral density	-0.032	0.014	-2.201	**0.028**

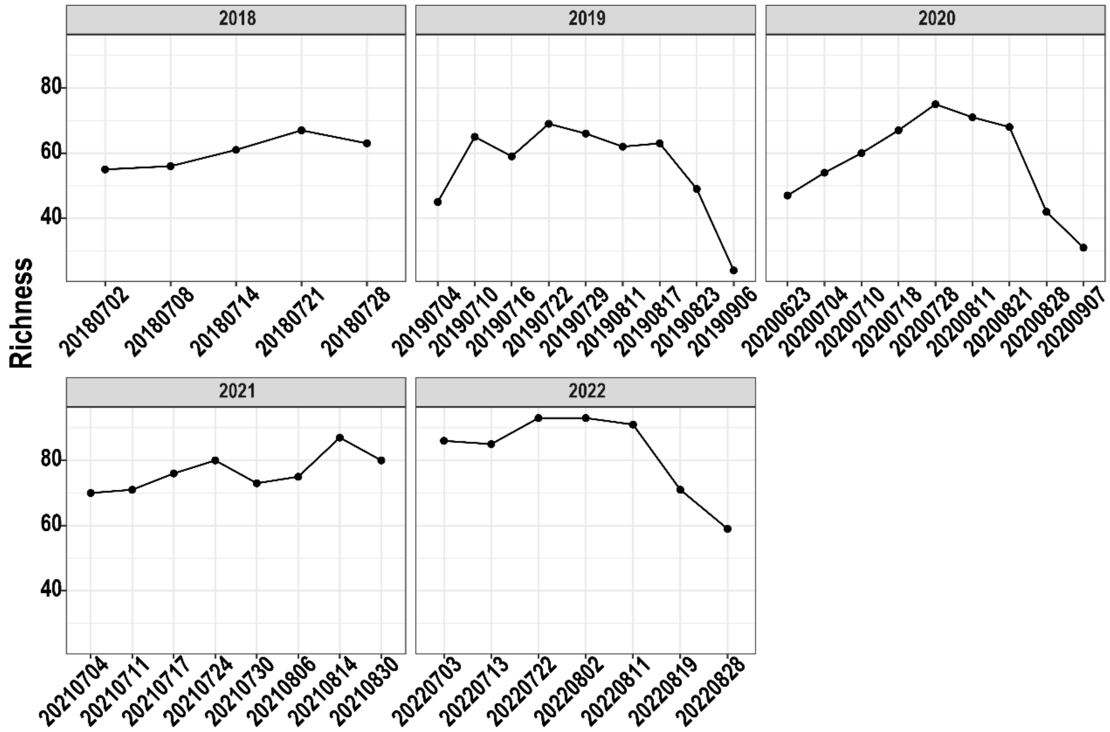

Fig. S1. Plant richness (the number of flowering species) recorded on each census day from 2018 to 2022.

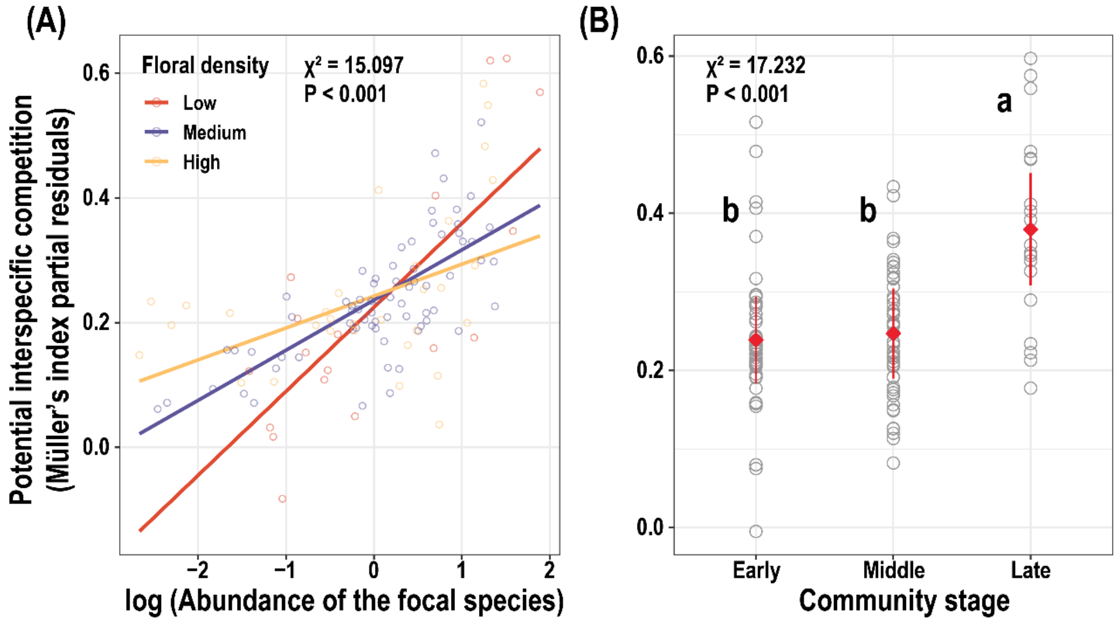

Fig. S2. Partial residuals plots showing the predicted values of (A) the interactive effect of abundance of the focal species and floral density, for low- (10th quantile; red dots and lines), medium- (50th quantile; blue dots and lines) and high (90th quantile; yellow dots and lines) floral density, and of (B) and (B) the effect of flowering period (early, middle and late) on the potential interspecific competition by only using the data from 2020 to 2022. The average Müller's index of acting bumblebee species for interspecific competition was used as the response variable. Floral density (n floral units/4m^2) and abundance of the acting bumblebee (n individuals) were log(x)- and z-transformed. For details on model estimates and significance, see Table S3.

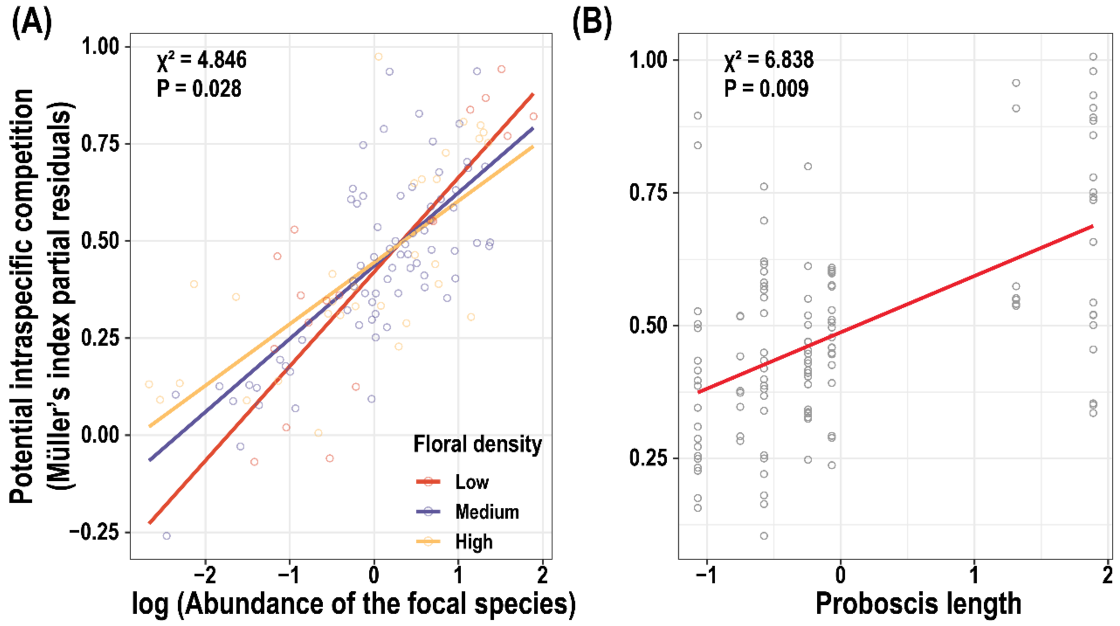

Fig. S3. Partial residuals plots showing the predicted values of (A) the interactive effect of abundance of the focal species and floral density, for low- (10th quantile; red dots and lines), medium- (50th quantile; blue dots and lines) and high (90th quantile; yellow dots and lines) floral density and (C) proboscis length on the potential intraspecific competition by only using the data from 2020 to 2022. The Müller's index for intraspecific competition was used as the response variable. Floral density (n floral units/4m^2) and abundance of the focal bumblebee (n individuals) were log(x)- and z-transformed. The proboscis length (in mm) was only z-transformed. For details on model estimates and significance, see Table S4.
